# Mechanistic Insights into the Stimulant Properties of Novel Psychoactive Substances (NPS) and Their Discrimination by the Dopamine Transporter—In Silico and In Vitro Exploration of Dissociative Diarylethylamines

**DOI:** 10.3390/brainsci8040063

**Published:** 2018-04-07

**Authors:** Michelle A. Sahai, Colin Davidson, Neelakshi Dutta, Jolanta Opacka-Juffry

**Affiliations:** 1 Department of Life Sciences, University of Roehampton, London SW15 4JD, UK; Michelle.Sahai@ROEHAMPTON.AC.UK; 2 St George’s, University of London, London SW17 0RE, UK; CDavidson2@uclan.ac.uk (C.D.); p0402670@sgul.ac.uk (N.D.); 3 Pharmacy & Biomedical Sciences, University of Central Lancashire, Preston PR1 2HE, UK

**Keywords:** dopamine, DAT, brain, addiction, molecular dynamics, free energy calculation, autoradiography, voltammetry, diphenidine

## Abstract

Novel psychoactive substances (NPS) may have unsuspected addiction potential through possessing stimulant properties. Stimulants normally act at the dopamine transporter (DAT) and thus increase dopamine (DA) availability in the brain, including nucleus accumbens, within the reward and addiction pathway. This paper aims to assess DAT responses to dissociative diarylethylamine NPS by means of in vitro and in silico approaches. We compared diphenidine (DPH) and 2-methoxydiphenidine (methoxphenidine, 2-MXP/MXP) for their binding to rat DAT, using autoradiography assessment of [^125^I]RTI-121 displacement in rat striatal sections. The drugs’ effects on electrically-evoked DA efflux were measured by means of fast cyclic voltammetry in rat accumbens slices. Computational modeling, molecular dynamics and alchemical free energy simulations were used to analyse the atomistic changes within DAT in response to each of the five dissociatives: DPH, 2-MXP, 3-MXP, 4-MXP and 2-Cl-DPH, and to calculate their relative binding free energy. DPH increased DA efflux as a result of its binding to DAT, whereas MXP had no significant effect on either DAT binding or evoked DA efflux. Our computational findings corroborate the above and explain the conformational responses and atomistic processes within DAT during its interactions with the dissociative NPS. We suggest DPH can have addictive liability, unlike MXP, despite the chemical similarities of these two NPS.

## 1. Introduction

A notable increase in the number of new psychoactive substances (NPS), formerly known as ‘legal highs’, ‘bath salts’ or ‘designer drugs’ ties in with the growing lines of evidence of their complex behavioural effects and health risks they may carry. NPS often resemble traditional drugs of abuse, although their pharmacological properties are unknown. Frequently, through minor chemical modification of the molecular structure of psychoactive drugs, different biological effects can be achieved, which includes the drug’s addictive liability. The latter links with the stimulant effects of drugs and involves the neurotransmitter dopamine. Stimulants have been known to raise dopamine availability in the brain, including the brain’s reward pathway that can be hijacked by stimulant drugs [1]. Perceptions of pleasure and reward are associated with the release of dopamine in the nucleus accumbens as part of the reward pathway; a similar phenomenon is also implicated in responses to stimulants whose repeated use may lead to drug dependence [2].

The molecular target for stimulants is the dopamine transporter (DAT), which can be obstructed by the drug that binds to it. DAT belongs to the Solute Carrier 6 gene family (*SLC6A3*) that encodes several Na^+^/Cl^−^-dependent neurotransmitter transporters (the NSS or neurotransmitter:sodium symporter family), including transporters for norepinephrine, serotonin, GABA and glycine [3]. NSS proteins function to couple the transport of Na^+^ down its concentration gradient with the uphill transport of the respective substrate. Additionally, several NSS proteins are characterized by co-transport of Cl^−^ [4]. DAT, like the other members of this family of transporters, has both an intracellular amino- and carboxyl-termini and twelve transmembrane (TM) helical domains [5,6,7]. The substrate binding site is known to be deeply buried in the transporter structure. Referred to as the S1 site, it has been resolved by X-ray crystallography [6,8,9] and previous computational modeling [10,11,12] to describe a site that overlaps with that of dopamine and many of the popular psychostimulants. It is also clearly distinct from the site observed for antidepressant binding (S2 site) to the leucine transporter (LeuT) which is found facing the extracellular vestibule nearly 11 Å above the S1 site [13].

A classical example of a drug that binds to DAT and obstructs the S1 site is cocaine [14]. Cocaine binding to the DAT reduces dopamine re-uptake from the extracellular compartment and leads to an increase in the synaptic concentrations of dopamine available for neurotransmission in the pathway affected (mesolimbic/mesocortical) [15]. Alternatively, drugs such as amphetamine, interact with the DAT when entering the presynaptic compartment where they displace newly synthesised dopamine [16], which also leads to an increase in basal dopamine concentrations in the synapse. The elevated concentration of extracellular (synaptic) DA underpins the behavioural response to stimulants, and with repeated use, may lead to drug dependence.

We have previously demonstrated a range of pro-dopaminergic effects among synthetic cathinones [17] and benzofurans [12,18] as studied by means of neurobiological methods in vitro, and also in silico, using molecular modeling of atomistic interactions between drugs and DAT [12]. By means of the in vitro fast cyclic voltammetry technique, we were able to estimate if the stimulant profile is more similar to that of amphetamine or cocaine. On the basis of radioligand binding studies we can visualise the drug’s binding at the dopamine transporter in the mesolimbic and mesocortical pathways of the brain, and we could say that some of the synthetic cathinones, such as bupropion, ethcathinone or diethylpropion do not act as a substrate releaser at the DAT. That allowed us to conclude that the wide-ranging pro-dopaminergic effects of cathinones were consistent with their different behavioural effects and popularity as recreational drugs [17]. Additionally, the varied and complex atomistic mechanisms of interactions between stimulants and DAT, which result in an increase in dopaminergic tone typical of stimulant drugs, can be studied by means of virtual methods of molecular modeling [12], which we apply in the present study to understand how relatively minor structural differences translate into different pharmacological NPS profiles of relevance to their addictive potential.

**Figure 1:**
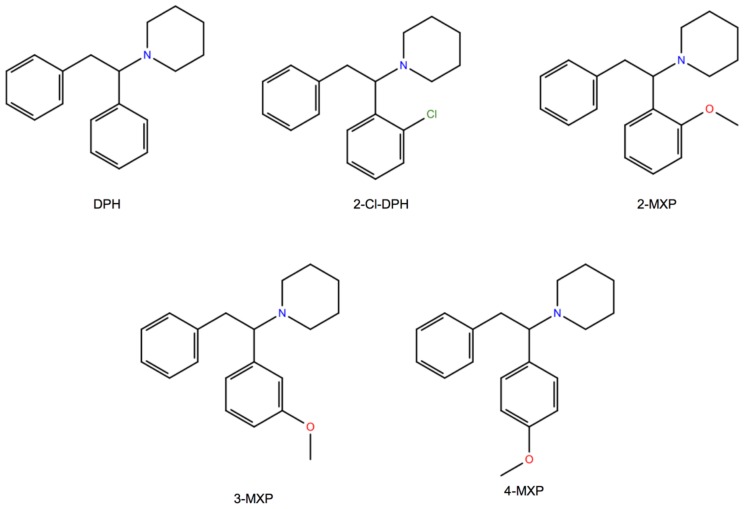
Molecular structures of diphenidine (DPH) and other aryl-substituted 1,2-diarylethylamines including 2-MeO-diphenidine (2-MXP), 3-MXP, 4-MXP and 2-Cl-DPH.

Here, we choose to study in silico a class of compounds that rigorously share the core of the 1,2-diarylethylamine structure and an ethylamine nucleus with aromatic substitutions (Figure 1). We also study two of them, the dissociative NPS, diphenidine (DPH) and its methoxylated derivative 2-methoxydiphenidine (methoxphenidine, 2-MXP/MXP), in vitro using the above neurobiological methods. DPH and 2-MXP replaced a ketamine-like drug methoxetamine (MXE) which was banned the UK in 2013; MXE was branded as a bladder-friendly ketamine, while ketamine has been associated with cystitis and bladder fibrosis [19,20].

It is worth noting that DPH and 2-MXP have been described as relatively selective *N*-Methyl-D-aspartate receptor (NMDA receptor or NMDAR) antagonists [21,22,23]. NMDA antagonism has been accepted to underpin the dissociative state effects that include sensory distortions and hallucinations, and depersonalization [19,24,25], although monoamine binding sites can also play a role in the psychoactive profiles on these drugs [25]. The recent interest in the pharmacology of these compounds is justified by the fact that both DPH and 2-MXP have been associated with adverse health effects including deaths [21,26,27,28,29].

Interestingly, DPH and 2-MXP have different functional potencies (IC_50_) at the dopamine transporter, 1.99 µM and 30 µM, respectively [22] and as confirmed in the recent study [30] where DPH was shown to be an inhibitor of the noradrenaline transporter (NET) (3.3 µM) and DAT (3.4 µM), while 2-MXP was mainly an inhibitor of the NET, with 7.8 µM inhibition compared to 65 µM at DAT . The monoamine transporter inhibition can contribute to their psychoactive properties and dictate addictive potential especially in the case of DPH which has a relatively high affinity (K_i_ = 0.23 µM) compared to 4.8 µM for 2-MXP at DAT [30].

Since this study relies heavily on rat in vitro data we endeavoured to be consistent in comparing the in silico data to the in vitro responses to psychostimulants in the same cellular background. As such, we utilised a previous homology model of the *Rattus norvegicus* dopamine transporter (rat DAT, rDAT) [12] to dock each of the five compounds (Figure 1). Their relative binding free energies were then calculated using alchemical free energy molecular dynamics simulations, particularly the free energy perturbation (FEP) method. The free energy predictions were subsequently compared with the experimental IC_50_ values that were reported earlier [22]. By using such in silico approaches we explored the possibility of predicting the DAT-binding properties, and thus addictive liability among this class of dissociative NPS. Awareness of addictive potential of NPS is important to both users and health services.

## 2. Methods

### 2.1. Animals

Eight week old male Wistar rats (Charles River, Harlow, UK) were kept on a 12/12 h light/dark cycle (lights on at 7 a.m.) with food and water *ad libitum*. Rats were treated in accordance with the U.K. Animals (Scientific Procedures) Act 1986 (related to the 1986 EU Directive 86/609/EEC) and sacrificed by cervical dislocation with no anaesthesia.

### 2.2. Reagents

All chemicals used were supplied by Sigma Chemicals (Poole, UK) except DPH and 2-MXP which were a gift from John Ramsey (TICTAC Communications Ltd., London, UK). The radioligand for the dopamine transporter, [^125^I]RTI-121 (specific activity 81.4 TBq/mmol) was purchased from Perkin Elmer (Beaconsfield, UK).

*Radioligand DAT binding study* was conducted as previously described [8]. Briefly, brains were removed and frozen at −40 °C in a mixture of methanol and dry ice, then stored at −80 °C. Frozen brains were cut into 20 µm serial coronal sections to harvest the striatum at +1.7 mm to −0.3 mm versus bregma [31], collected onto polysine-coated slides and stored at −80 °C. The autoradiography procedure was conducted according to Strazielle et al., 1998 [32]: preincubation in 0.05 M NaPB pH 7.4, incubation with 20 pM [^125^I]RTI-121 in NaPB pH 7.4 with increasing concentrations of the drugs tested (0–30 µM) for 60 min at room temperature; non-specific binding was assessed in the presence of 200 µM nomifensine. Slides were opposed to Kodak BioMax MR films for 4 days; autoradiograms were analysed using MCID™, Version 7.0, Imaging Research Inc. (St. Catharines, ON, Canada), *n* = 6 rats. Flat-field correction was applied. The striatal regions of interest were sampled in duplicates for relative optical density; left and right caudate values were averaged, and their means were calculated to assess the specific binding.

### 2.3. Fast Cyclic Voltammetry

*Carbon Fibre Microelectrodes*. Carbon fibre microelectrodes were constructed by inserting a single carbon fibre (Goodfellow Cambridge Ltd., Huntingdon, UK), 7 µm in diameter, into a 10 cm long borosilicate glass capillary tube. The capillary tube was then pulled using an electrode puller (P-30, Sutter Instruments Co., Novato, CA, USA) and the exposed carbon fibre was cut to approximately 70 µm under a microscope using a scalpel. Microelectrodes were backfilled with a saline solution before a length of copper wire was inserted into the end so it could be connected to the head-stage. A Ag/AgCl reference electrode and a steel wire auxiliary electrode were also connected to the head-stage and positioned within the recording chamber fluid, well away from the slice. Carbon fibre electrodes were calibrated using 5 or 10 µM dopamine in artificial cerebro-spinal fluid (aCSF).

*Fast cyclic voltammetry*. A triangular voltage waveform was applied to a carbon fibre microelectrode, which oxidises dopamine at ~600 mV. Calibrations of electrodes in a known concentration of dopamine allow the recorded Faradaic current to be converted into the relevant neurotransmitter concentration. Using a Millar Voltammetric Analyser (PD Systems, West Molesey, UK) we sampled dopamine at 8 Hz. Changes in the sampled signal were captured using a CED1401 micro3 analogue-to-digital converter (Cambridge Electronic Design (CED), Cambridge, UK), displayed using Spike2 v7.1 data capturing software.

*Electrical stimulation protocol*. Bipolar tungsten electrodes, with their tips 400 μm apart, were used to locally stimulate the core of the nucleus accumbens. Pseudo-one pulse stimulation was used to avoid the activation of autoreceptors [33] which occurs approximately 500 ms after striatal dopamine release [34,35]. A train of 10 × 1 ms 10 mA pulses at 100 Hz was applied every 5 min using a Neurolog NL800 stimulus isolator (Warner Instruments, Hamden, CT, USA) under computer control (Spike, CED, Cambridge, UK).

*Experimental protocol*. To begin an experiment, slices were transferred from the slice saver to a laminar flow recording chamber that was supplied with aCSF via gravity feed, at a rate of 100 mL/h. Slices were left to equilibrate in the recording chamber for 30 min before starting stimulation of the slice, and the tips of both stimulating and recording electrodes were placed in the accumbens to record monoamine release. Recording took place from the beginning of this 30 min period as large spontaneous release events of dopamine can occur, which is indicative of poor slice health [36], and on such occasions (5–10%) the experiments were terminated.

### 2.4. Statistics

DAT binding data were analysed with a 1-way ANOVA. Voltammetry data were analysed for peak effects of each drug concentration on each brain slice, which was typically found around 45–50 min after drug application. Statistical analysis was carried out using SigmaPlot (v. 11.0), a 1-way ANOVA (independent variable = concentration) was used with post-hoc Tukey’s. In all graphs data are presented as means ± SEMs and significance is set at *p* < 0.05.

### 2.5. Computational System Setup

*Modeling of the Rattus norvegicus dopamine transporter (rDAT).* The construction and refinement of the homology model of the *Rattus norvegicus* dopamine (rDAT) transporter has been previously reported [12] using established protocols used in the construction of a human DAT (hDAT) model [37,38,39,40]. Briefly, we used Modeller 9v17 [41] and the previously published sequence alignment of the NSS family of proteins to first construct the transmembrane (TM) part of the rDAT (residues 57–589) based on the recent crystal structure of the *Drosophila melanogaster* dopamine transporter (dDAT) bound to dopamine (PDB ID: 4XP1) [9]. An adaptation of this sequence alignment, created by the Alignment-Annotator web server [42], is provided in Figure S1—in the Supplementary Material for convenience. The newly crystallized dDAT structure is well suited as a template for homology modeling of rDAT because the overall sequence identity is >50% [6], with the sequence identity between the TM segments of rDAT and dDAT being 61%, and having a Root Mean Square Deviation (RMSD) of <1 Å for the critical regions of the binding site and ion binding sites, TMs 1, 6 and 8 [12], which are key to the inferences we describe herein.

For completion, we also used the sequence alignment in Figure S1 and the N- and C-terminal regions modelled for hDAT from ab initio methods [40] to include Modeller 9v17 [41] homology models of the terminal domains for rDAT. Based on the align module of Modeller 9v17 [41], two functional Na^+^ and one Cl^−^ ion in 4XP1 were also added to the S1 binding site. PROPKA [43] was used to determine the protonated state of the ionizable Glu490 residue of rDAT while a disulfide bond was introduced in EL2, between Cys180 and Cys189.

*Compound preparation and docking.* The chemical structures in the (S)-enantiomer of the five compounds (Figure 1) were built using the LigPrep module of Schrödinger Release 2017-1 [44] with the OPLS3 force field. The prepared compounds all carried a net positive charge as assigned by Epik [44]. The rDAT homology model was prepared using the Protein Preparation Wizard module in Maestro, following which the Induced fit docking (IFD) protocol, in the Schrödinger software suite was implemented to dock 2-MXP (Figure 1) to the homology model of rDAT. Since 2-MXP is one of the largest of the five compounds it was used to create a greater volume in the binding site, that was subsequently replaced by the other compounds. The residues Phe76, Asp79, Ser149, Val152, Tyr156, Asn157, Phe326, Val328 and Ser422, previously identified as important for binding psychostimulants of comparable size [10,11], were used to define the docking grid box. Dockings was then performed using standard precision (SP). Random initial positions and conformations of the ligand were screened for clashes with the protein and subsequently refined by allowing flexibility of the side-chains in the binding site.

The bound 2-MXP was then replaced with the other compounds to create the corresponding complexes (rDAT-DPH, rDAT-CLD, rDAT-3-MXP and rDAT-4-MXP) by aligning the backbone atoms to overlap the binding positions, while maintaining the amino (NH) group interaction with Asp 79. In the absence of a crystal structure of DAT with these compounds we hypothesised that the best binding pose for these compounds would initially exploit the key interactions of residues Phe76, Asp79, Ser149, Val152, Tyr156, Asn157, Phe325, Val327 and Ser421, as seen in the crystal structures of dopamine, amphetamine, MDMA and cocaine [9] and which was also validated and exploited for docking 5-MAPB and 5-APB in a previous study [12]. The force-field parameters for the compounds were obtained from the Acellera small molecule parameterization tool implemented in the HTMD 1.11.2 suite [45].

### 2.6. Molecular Dynamics Simulations

The rDAT complexes (rDAT-DPH, rDAT-CLD, rDAT-2-MXP, rDAT-3-MXP and rDAT-4-MXP) were immersed in biophysically relevant membrane environments, a mixture of POPE/POPC/PIP_2_/POPS/cholesterol, closely resembling the neuronal cell plasma membrane, with explicit water, internal ions and added salts as described previously. See the earlier work for details of the protocol [12].

For all complexes a previously described multistep equilibration protocol [46] was performed with the NAMD software, version 2.9 [47] to remove the close contacts in the structure, the backbones were initially fixed and then harmonically constrained, and water was restrained by small forces from penetrating the protein-lipid interface. The constraints on the protein were released gradually in three steps of 300 ps each, changing the force constants from 1 to 0.5, and 0.1 kcal/(mol Å2), respectively, with a time step of 1 fs. This was then followed by a short (100 ns) unbiased MD simulation performed with a 2 fs integration time-step and under constant temperature (310 K) maintained with Langevin dynamics, and 1 atm constant pressure achieved by using the hybrid Nosé-Hoover Langevin piston method on a flexible periodic cell to capture long-range effects. The simulated system, including the transporter embedded in a membrane patch and water layers on each side containing Na^+^ and Cl^−^ ions (corresponding to a concentration of 150 mM NaCl), was composed of approximately 266,590 atoms in a box with the final dimensions of 138 × 146 × 153 Å.

After this equilibration phase, unbiased production MD simulations were carried out using GPUS and the ACEMD software [48] with an established protocol [12,39] for a further 300 ns. Briefly, the simulations employed the all-atom CHARMM27 force field for proteins with CMAP corrections [49] as well as the CHARMM36 force field for lipids [50], the TIP3P water model, and the CHARMM-compatible force-field parameter set for PIP_2_ lipids [51]. The PME method for electrostatic calculations was used, along with 4 fs integration time-step with standard mass repartitioning procedure for hydrogen atoms implemented in ACEMD. More details about the computational protocol can be found in (Khelashvili et al., 2015b). The trajectories were analysed with the R software [52] and VMD [53] for graphical representation.

In total, at least 3 µs of simulation time including the MD runs and the following free energy perturbation (FEP) calculations were accumulated.

### 2.7. Free Energy Perturbation Calculations

**Figure 2:**
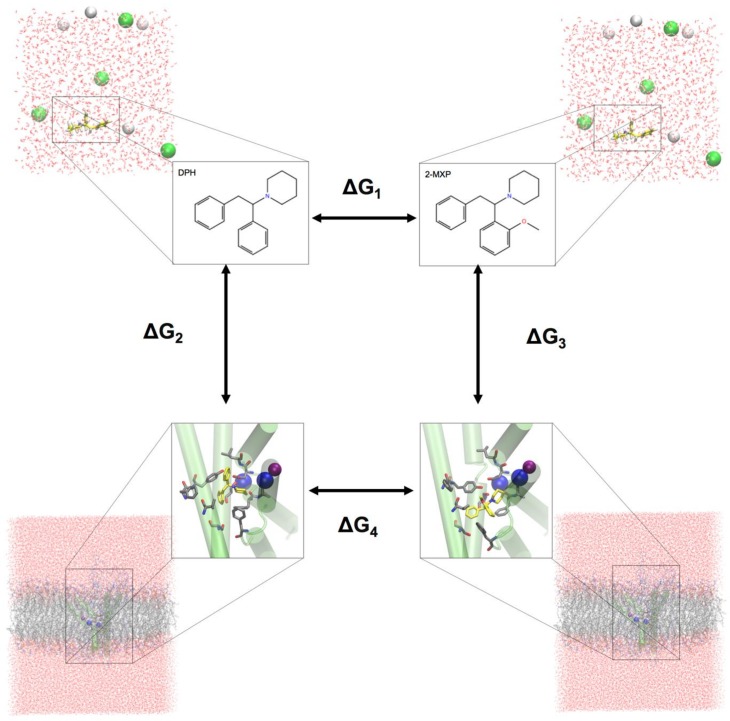
The thermodynamic perturbation cycle between the compounds DPH and 2-MXP. A similar cycle would apply to the free energy change from DPH to the other three compounds (2-Cl-DPH, 3-MXP and 4-MXP). Here we highlight the complexity of the full systems in each step of the transformation as well as the components (compounds, protein, ions and membrane) that are boxed. ∆G_1_ represents the unbound solvent (ligand in water) state while ∆G_4_ represents the bound (protein-ligand complex in water) state.

To evaluate the change in the binding free energy of the four compounds (2-Cl-DPH, 2-MXP, 3-MXP and 4-MXP) from DPH, we utilized a two-step free energy calculation approach. As an example, we show in Figure 2 the change in the binding free energy between compounds DPH and 2-MXP. This can be calculated by either ∆G_4_ − ∆G_1_ or ∆G_3_ − ∆G_2_. However, the change from ∆G_4_ − ∆G_1_ is computationally more practical. ∆G_1_ represents the unbound solvent (ligand in water) state while ∆G_4_ represents the bound (protein-ligand complex in water) state. We are calculating the relative binding affinity of two compounds rather than the absolute protein-ligand binding free energy calculation which is more challenging as the introduction of the protein adds significantly more degrees of freedom to the system [54].

Two independent transformations between each of the four compounds (2-Cl-DPH, 2-MXP, 3-MXP and 4-MXP) and DPH were calculated in both a water-solvent environment with both the protein (and membrane) present and absent, respectively. The free energy change ∆G of each perturbation was evaluated by the alchemical free-energy perturbation (FEP) approach [55,56] that has been applied previously to de novo mutations in the human dopamine transporter [37,38].

The free energy perturbation procedure with a softcore potential implementation was carried out with the NAMD software version 2.12 [47] and with the same simulation systems with explicit solvent as described above. For the FEP computation, the coupling parameter λ varied from 0 to 1 such that each window did not exceed 3 kcal/mol for a total of 400 ps for the full transformation. In the hysteresis tests the results differed from the annihilation in the same interval by ~1 kcal/mol. Each reported value is the average of at least three runs starting from different points (after at least 100 ns) of the MD trajectories. Table S1 of the Supplemental Information gives the details of the bound (protein-ligand complex in water) state or ∆G_4_. Using the restraining potential approach (Wang et al., 2006), a potential representing the interaction of the atoms being annihilated with the binding residues, including the crucial salt bridges between the N1 atom of each compound and the CG of Asp 79 and OH of Tyr 156 in the S1 site were applied [10,11]. The final solvation energies were calculated as the algebraic sum of the FEP and restraining energy values. A similar protocol has been described elsewhere [37,46,57].

The aqueous solvation energy of the ligand in the simulation system was calculated for direct comparison in a 30 × 30 × 30 Å^3^ water box containing two Na^+^ ions and one Cl^−^ ion (equivalent to 120 mM NaCl) using exactly the same FEP/MD procedure as above but without restraints.

## 3. Results

**Figure 3:**
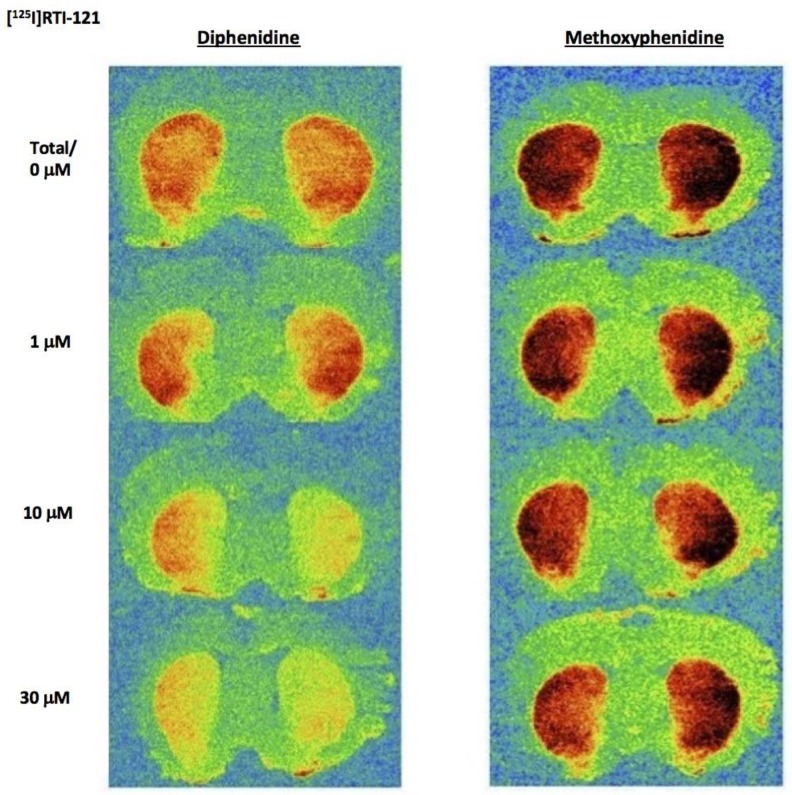
Representative autoradiograms showing the binding of the radioligand [^125^I]RTI-121 in the brain striatal sections in the presence of increasing concentrations of DPH and 2-MXP (MXP).

**Figure 4:**
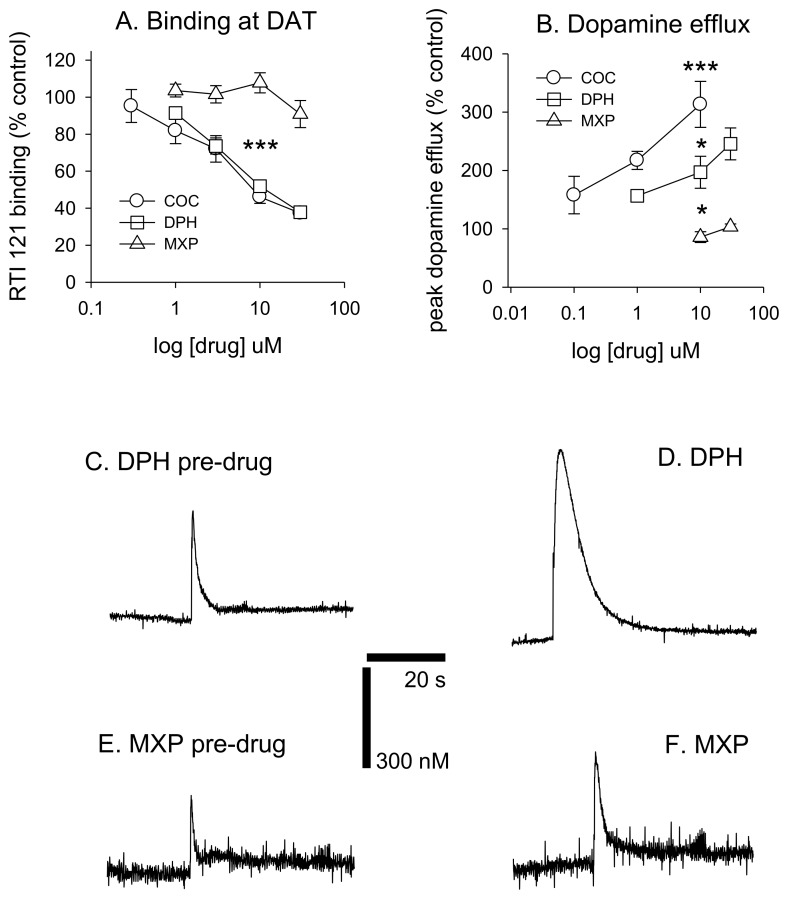
Top panel (**A,B**): Comparative effects of DPH, 2-MXP (MXP) and cocaine-quantitative in vitro data: (**A**) Specific DAT binding expressed as % of [^125^I]RTI-121 binding; *n* = 6 per drug concentration. (**B**) peak DA efflux as measured by means of fast cycling voltammetry *n* = 4–7. Bottom panels (**C**–**F**): Representative dopamine efflux events after brief electrical stimulation for DPH and 2-MXP (10 pulses at 100 Hz). (**C**,**E**) show control dopamine efflux prior to drug application. (**D**,**F**) show dopamine efflux 60 min after drug administration. 60 s of data is shown for each trace and peak dopamine efflux is ~250 nM under control conditions. DAT: dopamine transporter; COC: cocaine; DPH: diphenidine; MXP: methoxyphenidine. Values are means ± SEM. Panel A: *** *p* < 0.001 for both COC and DPH vs. MXP at 10 µM. Panel B: * *p* < 0.05 for COC vs. DPH and for DPH vs. MXP, *** *p* < 0.001 for COC vs. MXP, all for 10 µM drug concentrations. For drug concentrations vs. control statistics see text.

Diphenidine displaced [^125^I]RTI-121 (RTI) binding in a concentration-dependent manner (*F*(4, 29) = 33.26, *p* < 0.001) with 3, 10 and 30 μM causing a significant reduction in RTI binding (*p* < 0.05) (Figure 3 and Figure 4A). There was no effect of methoxphenidine on RTI binding (*F*(4, 29) = 1.47, *p* = 0.24). Cocaine was also tested for comparison (autoradiograms not shown); cocaine displaced RTI binding in a concentration-dependent manner (*F*(5, 35) = 18.701, *p* < 0.001. Tukey’s test revealed that 3, 10 and 30 μM cocaine significantly reduced RTI binding (all *p* < 0.05). A 1-way ANOVA was used to compare the 3 drugs at 10 μM (the only common concentration used in the dopamine efflux experiments) and there was a significant difference between the 3 drugs (*F*(2, 17) = 72.683, *p* < 0.001. Tukey’s showed that both cocaine and diphenidine caused a significant reduction in RTI binding vs. methoxyphenidine (Figure 4A).

DPH also increased peak dopamine efflux after electrical stimulation (*F*(3, 19) = 14.405, *p* < 0.001, with 10 and 30 μM significantly increasing dopamine efflux vs. controls (both *p* < 0.05). 2-MXP had no significant effect on peak dopamine efflux *F*(2, 15) = 1.91, *p* = 0.187. MXP voltammetry was only tested at 10 and 30 μM. Cocaine also increased dopamine efflux (*F*(3, 21) = 14.907, *p* < 0.001). Tukey’s revealed that both 1 and 10 μM significantly increased dopamine efflux (both *p* < 0.05). We also compared drugs at 10 μM using a 1-way ANOVA; *F*(2, 14) = 19.884, *p* < 0.001) with post-hoc Tukey’s revealing that cocaine had a significantly greater effect on dopamine efflux vs. both diphenidine (*p* < 0.05) and methoxyphenidine (*p* < 0.001) and diphenidine had a significantly greater effect on dopamine efflux vs. methoxyphendine (*p* < 0.05). See Figure 4B. Dopamine efflux events after brief electrical stimulation (10 pulses at 100 Hz) are shown in Figure 4C–F, demonstrating the differences between DPH and 2-MHP in their respective abilities to increase dopamine levels at 30 μM.

### 3.1. Relative Binding Free Energies

The binding free energy change from DPH to its aryl-substituted 1,2-diarylethylamines analogs (Figure 1) are calculated and compared with the previously determined experimental IC_50_ values from rDAT (Table 1) [22]. Free energy methods have been shown to have good chemical accuracy [56,58] in predicting the binding free energy change (∆∆G), which is the cost in free energy of substituting one compound with another compound. This is usually in combination with well-tuned force fields that have reasonable agreement with experimental results, where the RMSD in hydration free energy of small organic molecules between calculated and experiment are ~1.0 kcal/mol using the more common force fields like CHARMM. Therefore, the hypothetical binding poses for each compound as described above, which is shared by many other compounds in the binding site should give the best correlation between the predicted binding free energy and the experimentally measured binding affinity IC_50_ values. A positive value would suggest a cost in energy, thus the substituted compound should be less favourable. Conversely, a negative value means the substituent is more favourable.

Based on the initial rDAT complexes constructed by docking, MD simulations were performed to incorporate conformational flexibility into both rDAT and the corresponding ligands (2-MXP, 3-MXP, 4-MXP, DPH, CLD) to assess the persistence of the key interactions in the S1 site (Phe76, Asp79, Ser149, Val152, Tyr156, Asn157, Phe326, Val328 and Ser422). As such, the monitored Root Mean Square Deviation (RMSD) of the transmembrane regions of rDAT showed that the RMSDs reached equilibration within 100 ns simulation for each complex (Figure S2A). Structural superimposition of the docking pose and a representative snapshot from the MD simulations for the five compounds in the rDAT binding site indicates some flexibility (e.g., the RMSDs of DPH and 2-MXP before and after MD simulation were 0.56 Å and 0.75 Å, respectively (Figure S2B)), but the key interactions such as the salt bridge between the protonated nitrogen of ligand and the Asp79 was preserved. As such, snapshots were taken at 100 ns and subsequently at 150 ns and 175 ns to further estimate the molecular basis of 2-MXP, 3-MXP, 4-MXP, DPH and CLD binding specificity to rDAT via the FEP method.

**Figure 5:**
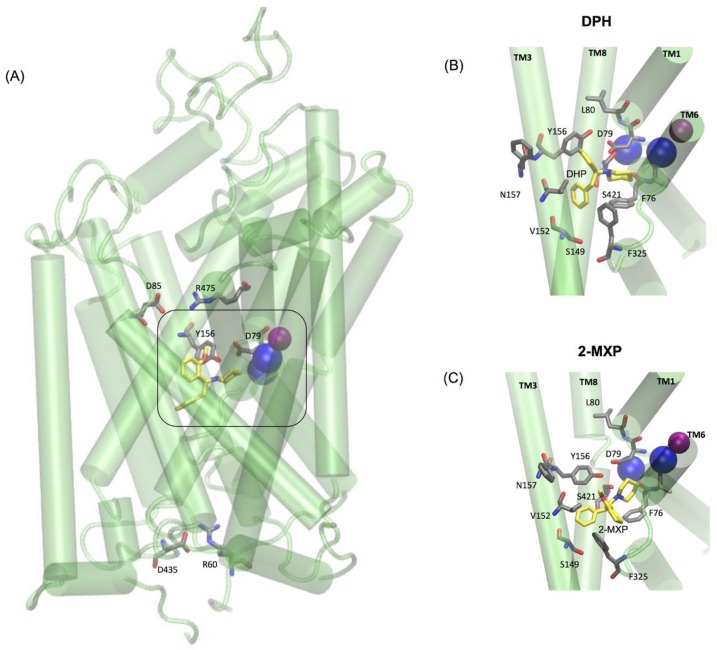
(**A**) Simplified representation of the molecular model of rDAT and the positions of the two oppositely charged pairs of residues, Arg 85–Asp 475 and Arg 60–Asp 435, functioning as the extracellular (EC) and intracellular (IC) gates respectively and the Asp79 and Tyr156 residues shown to be important in stabilizing the S1 binding pocket (boxed) via a hydrogen bond. rDAT in complex with (**B**) DPH (yellow) and (**C**) 2-MXP (yellow). Each of these distinct compounds occupies a binding pocket that is deeply buried in the transporter structure. Selected central binding site residues from each compound are shown in grey and labelled respectively. Additionally, blue and purple spheres represent the internal sodium and chloride ions, respectively.

The two oppositely charged pairs of residues, Arg 85–Asp 475 and Arg 60–Asp 435, functioning as the extracellular (EC) and intracellular (IC) gates respectively, were maintained as well as the stabilizing hydrogen bond between Asp79 and Tyr156 shown to be important in stabilizing the S1 binding pocket [10,12] (Figure 5A). Additionally, as previously mentioned the binding pose for each compound was aligned so that they maintained the amine (NH) group interaction with Asp 79. For the purpose of the free-energy calculations this was aided by the use of distance restraints between the crucial salt bridge of the N1 atom of each compound and the CG of Asp 79 and OH of Tyr 156.

Interestingly, the values for ∆∆G reveal ranked predictive binding free energies with respect to DPH as follows 4-MXP (−0.75 kcal/mol) > 3-MXP (−0.65 kcal/mol) > DPH (0 kcal/mol) > 2-Cl-DPH (0.35 kcal/mol) > 2-MXP (3.63 kcal/mol).

The ∆∆G values from DPH to 2-Cl-DPH (0.35 kcal/mol) and DPH to 2-MXP (3.63 kcal/mol) are positive indicating unfavourable substitutions, with the most unfavourable being 2-MXP. This is in agreement with the IC_50_ values whereby 2-MXP (30 µM) is less potent at rDAT compared to 2-Cl-DPH (10.5 µM).

The measured IC_50_ values for 3-MXP (0.587 µM) and 4-MXP (2.23 µM) while showing unfavourable changes are quite close to DPH (1.99 µM). While the change in binding free energies (∆∆G) are small, they suggest favourable substitutions for the methoxy group in 3-MXP (−0.65 kcal/mol) and 4-MXP (−0.75 kcal/mol). With 4-MXP ranking slightly more favourable than 3-MXP, which is in disagreement with experimental data as the IC_50_ values show that 4-MXP is more potent.

### 3.2. Molecular Dynamics Simulations

The extended molecular dynamics simulations also revealed some mechanistic insights into the different binding properties of each compound. The simulations reveal a number of concerted motions not only with the IC and EC gates but also distances between the Na1 and Na2 ions and the Asp 79 and Tyr 156 hydrogen bond. We also characterise the IC movements based on the concerted motions of the intracellular transmembrane TM1a, TM6b, and TM9 segments and additional movements on the extracellular side by the TM1b and TM6a segments, previously identified as markers for opening of the intracellular vestibule [39].

**Figure 6:**
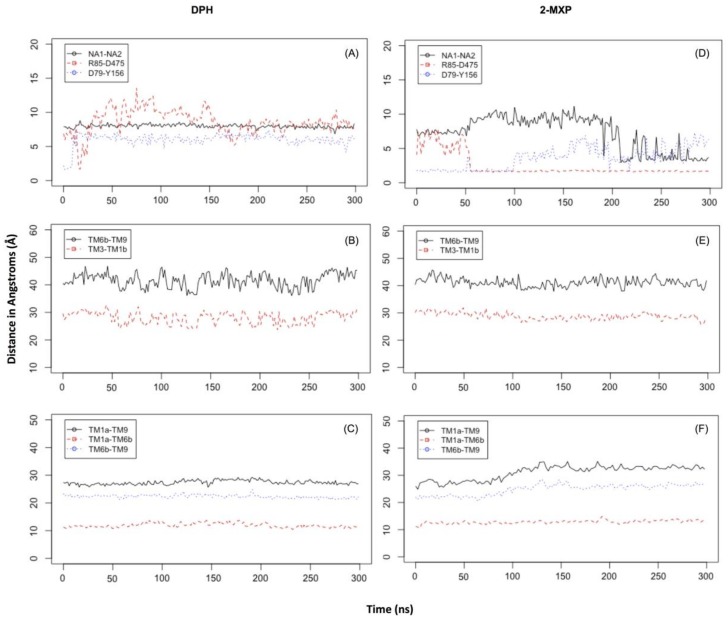

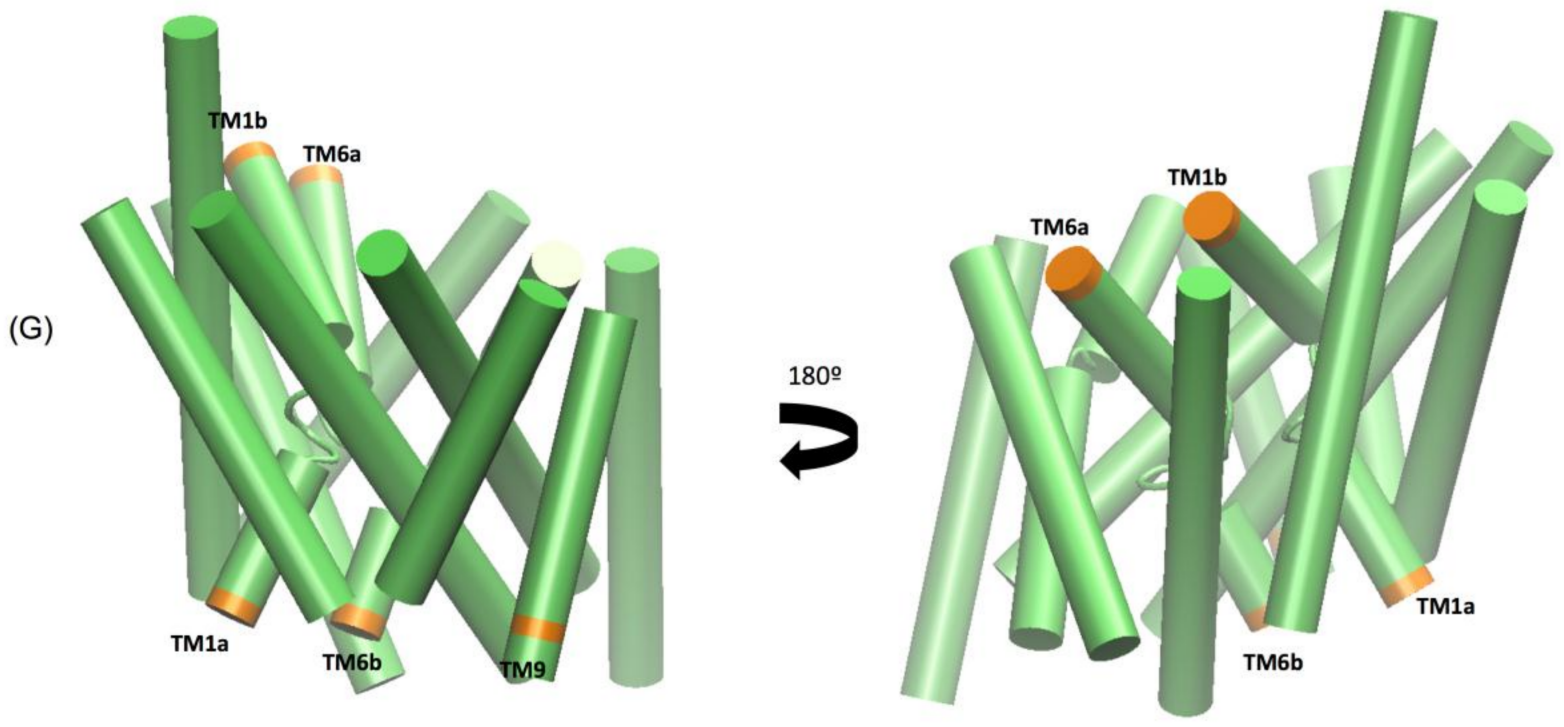
Time evolution in the simulations for rDAT when bound to DPH (**A**–**C**) and 2-MXP (**D**–**F**). (**A**,**D**) depict the distances between D79 and Y156, Na1 and Na2 and R85 and D475. (**B**,**C**,**E**,**F**) panels depict the Cβ-Cβ distances between residues in various extracellular and intracellular transmembrane (TM) segments respectively for I67 (in TM1a) and L446 (in TM9), I67 (in TM1a) and S332 (in TM6b) and S332 (in TM6b) and L446 (in TM9); E306 (in TM6a) and F171 (in TM3); and F171 (in TM3) and K92 (in TM1b). (G) The IC and EC segments in the protein TM regions from the middle and bottom panels are labelled and coloured in orange.

In DPH we see a disruption of the extracellular network with the ionic interaction between Arg 85 and Asp 475 for DPH (a feature not seen with the other compounds) (Figure 6A). This was complemented by the movements seen in the extracellular TM6b-TM9 and TM3-TM1b segments (Figure 6B). This suggests a mechanism involving a conformational change whereby DAT opens extracellularly. There is no evidence of Na2 ion destabilization or disruption of the hydrogen bond between Asp79 and Tyr156 during these timescales (Figure 6A) or any effect on the intracellular TM segments, TM1a-TM6b, TM1a-TM9, and TM6b-TM9 (Figure 6C).

The simulations reveal that 2-MXP changes position in the rDAT binding site in such a way that its electron-donating, 2-methoxy (-CH_3_O) group, that is also an ortho-substitution, is oriented away from the electron-donating phenolic-OH on Y156 (Figure 5C). Here we observe not only destabilization of the Na2 ion (Figure 6D) but also isomerization to the inward-facing state (as monitored by the movements in the intracellular TM segments—Figure 6F). There is no evidence of disruption of the extracellular network, instead the ionic interaction between Arg 85 and Asp 475 becomes more stable (Figure 6E).

At these timescales there is some evidence of isomerization with respect to 2-Cl-DPH at rDAT (Figure S1—in the Supplemental Material) as seen in Na1 and Na2 distance change (Figure S3A) and the increase in distances in the intracellular TM segments (Figure S3C). The extracellular network remains intact as well as the D79-Y156 hydrogen bond (Figures S3A and S3B). 2-Cl-DPH also changes position (tilts) in the binding site but it also contains an electron withdrawing chloride in the ortho-position, giving rise to an electron-poor ring substitution which could also explain its unfavourable IC_50_ and ∆∆G values.

With respect to 3-MXP and 4-MXP, the -CH_3_O group substitutions are classified as meta- and para- aromatic substitutions, respectively (Figures S4 and S5). Since we know that chemically the -CH3O group is described as an activator or electron donor to the ring at these positions this could potentially explain the more favourable ∆∆G values compared to 2-Cl-DPH and 2-MXP. Similarly, to 2-MXP, 4-MXP not only displays destabilization of the Na2 ion but also isomerization to the inward-facing state (Figure S5A,C). There is also no evidence of disruption of the extracellular network, instead the ionic interaction between Arg 85 and Asp 475 becomes much more stable (Figure S5B). At these timescales, 3-MXP does display evidence of isomerization to the inward-facing state (Figure S4C), with increased minimum distances changing between the intracellular TM segments, TM1a-TM6b, TM1a-TM9, and TM6b-TM9 but there is no change in the distance between Na1 and Na2 (Figure 4A) or the extracellular network (Figure 4B). Longer simulations are needed to fully explore these changes.

## 4. Discussion

In the present study, we analysed the mechanism of binding between rat DAT and aryl-substituted 1,2-diarylethylamines using docking and alchemical free energy approaches in conjunction with in vitro neurobiological experimental measurements, which we applied to 2 diarylethylamine NPS of that class, diphenidine and methoxphenidine. While DPH and 2-MXP exert their dissociative effects via *N*-methyl-D-aspartate receptor antagonism [22], their psychoactive effects can also be influenced by their binding to the monoamine transporters, with DPH and 2-MXP having their highest affinity for the DAT, followed by the noradrenaline transporter (NET) and serotonin transporter (SERT) [30]. DPH and 2-MXP have markedly different functional potencies at DAT, as represented by their IC_50_ values of 1.99 µM and 30 µM for DPH and 2-MXP, respectively, at human DAT stably expressed in HEK293 cells [22]. For the sake of comparison, the IC_50_ of cocaine as the most potent stimulant ranges from 0.4 µM to 1.3 µM at DAT expressed in transfected HEK293 cells, as published in the literature [59].

Our neurobiological findings suggest that DPH significantly increased dopamine efflux, as a result of binding to DAT, demonstrated in the competition with 125-iodine labelled RTI-121 in rat brain sections, whereas 2-MXP had no significant effect on either RTI-121 binding or evoked dopamine efflux in the nucleus accumbens in vitro. Interestingly, despite displacing RTI-121 binding with similar potency to cocaine, DPH had a significantly lesser effect on dopamine efflux as measured using fast cyclic voltammetry. These data, consistent with the IC_50_ differences reported by Wallach et al., 2016, suggest that DPH exhibits pro-dopaminergic stimulant-type effects in the brain tissue, and therefore this NPS can have some addictive liability. In addition to suggesting that DPH might have addictive liability due to its effects on dopamine efflux, we might also tentatively highlight its potential utility as a pharmacotherapy. Dopamine transporter ligands might be useful as substitution or maintenance treatments for psychostimulant abuse [60] or as treatments for attention deficit hyperactivity disorder (ADHD) or even as treatments for depression, for example bupropion. Given DPH is also an antagonist at NMDA receptors, as well as a DAT blocker, it may even have potential as a fast-acting antidepressant as seen with ketamine [61]. We have previously reviewed the potential use of novel psychoactive substances, including dissociatives, such as DPH, in CNS disorders [62].

On the contrary, 2-MXP, despite its apparent chemical similarity to DPH, does not exhibit pro-dopaminergic stimulant effects as assessed in the brain tissue in vitro. It is most interesting to understand why subtle structural differences result in such varied stimulant features of novel psychoactive substances (NPS) and what basic atomistic interactions of DAT as the biological target for stimulants decide about the discrimination of potential stimulants at DAT.

Therefore, we calculated relative binding free energy of the analogs 2-Cl-DPH and MXP. That exercise showed unfavourable substitutions when compared to DPH, in agreement with the experimental IC_50_ values, and consistent with our in vitro comparison of the pro-dopaminergic effects of DPH and MXP. While, the calculations using two other compounds 3-MXP and 4-MXP reveal favourable substitutions, although their IC_50_ values from Wallach et al. [12] show small unfavourable changes compared to DPH. Conformational changes emerging over long-scale simulations have also indicated different structural and dynamic elements of the mechanisms governing the interactions of these various compounds with DAT. Structural rearrangements in DAT when bound to DPH show evidence of an outward facing conformation, specifically the rearrangement of the extracellular gates, a well-known conformation adopted by inhibitors like cocaine [10,11,63,64]. This observation is supported by the present in vitro data where DPH acts as a DAT inhibitor but weaker than cocaine, which can cause a 4-fold increase in DA efflux, as previously observed under identical conditions [65]. In contrast when 2-MXP is bound to DAT, it shows evidence of adopting an inward facing conformation of DAT, such as the spontaneous release of Na2 and the rearrangement of the intracellular gates. Although this is a conformation adopted by many substrate releasers like amphetamine and 5-MAPB [12], 2-MXP does not appear to be a DAT inhibitor nor does it reveal reverse transport at the concentrations used in this study (up to 30 μM; data not shown) (Figure 4—bottom panel). Longer atomistic simulations are needed to resolve the rearrangements seen for 2-MXP as well as 2-Cl-DPH, 3-MXP and 4-MXP that also show stabilisation of the inward facing conformation of DAT.

While the free energy pertubation method itself is a good measure of the binding affinity between two molecules, it does highlight some considerations for future studies, including the size and position of the substitution. Most importantly, the geometric and dynamic properties of a modelled protein-ligand complex contribute entropically to the binding mode of ligands. Thus, it is still challenging to rank with chemical accuracy a series of ligand analogues in a consistent way for systems where there are considerable fluctuations in the binding mode. In this study we face this issue calculating relative binding free energies of the aryl-substituted 1,2-diarylethylamines analogs because of the presence of multiple metastable ligand orientations which can cause convergence problems. Distance restraints were utilised but the use of orientational restraints may further accelerate the convergence of these calculations [66]. We aim to further investigate this with other classes of NPS with different scaffolds and substitutions. Calculating the free-energy of binding from different snapshots of long atomistic-simulations may also reveal the energy change associated with binding pose changes.

This study also demonstrates the ability of the alchemical free energy approach in combination with docking and homology modeling to be an effective means of investigating and characterising novel psychoactive substances. Additionally, these methods highlight that a structural analysis is important when creating a stimulant profile as it adds to the insights of binding and functional data derived from functional studies applied in animal models. To our best knowledge, this is the first application of relative binding free energy calculation in the studies on the biological effects of NPS, and similar approaches are plausible with some other biological targets, which would expand our understanding of stimulant mechanisms of NPS and other drugs of addiction, as psychoactive effects of drugs can be influenced by their binding to other monoamine transporters, namely NET and/or SERT. It is important to understand the molecular determinants of stimulant actions which may underlie their distinct pharmacological effects at DAT and also at other transporters like SERT and NET as well as species effects.

This study considered the responses of both in vitro data and in silico data to psychostimulants in the same cellular background, rDAT. A previous study by Dawn Han and Howard Gu show that when popular psychostimulant drugs like cocaine, methylphenidate, amphetamine, methamphetamine and MDMA are tested for their relative affinities, their sensitivities at the human and mouse transporters were similar (Ki values are within 4-fold) [67]. Therefore, there is translational significance to this study supporting the use of rodent models to represent pharmacological, functional and now structural changes of psychostimulants when acting in humans. This is not surprising because rDAT and mDAT share 99% sequence similarity and identity to hDAT. Nevertheless, there is evidence and reason to investigate in detail species effects that could be driving potential differences like abolished reward mechanisms through mutations in mDAT and hDAT [68,69] in further publications and from longer simulations.

To conclude, our in vitro study with the brain tissue indicates that diphenidine can increase dopamine efflux as a result of its binding to dopamine transporter, whereas methoxphenidine has no significant effect on either RTI-121 binding or evoked dopamine efflux. These data suggest that diphenidine can have some addictive liability, unlike methoxphenidine, despite the chemical similarities of these two NPS.

The present computational study supports the neurobiological findings of DPH when compared with 2-MXP, and provides novel insights into the mechanisms that underpin the pro-dopaminergic, hence stimulant, interactions between NPS and DAT. Longer atomistic simulations are needed to confidently resolve whether the DPH stimulant behaves in a cocaine-like manner in terms of its effects on the conformational rearrangements within the DAT. While this novel in silico approach informs about the potential addictive effects of a prevalent dissociative NPS, diphenidine, it also tackles the core physical mechanisms that decide about addictive properties of other NPS, of direct relevance to the health risks linked with their use.

## Figures and Tables

**Table 1:** Calculated binding free energy change (∆∆G, kcal/mol) of the substituted analogs against DPH and their experimental IC_50_ values (µM)* from Wallach et al., 2016. ∆G_1_ represents the unbound solvent (ligand in water) state while ∆G_4_ represents the bound (protein-ligand complex in water) state.

Compound	∆G_1_ w.r.t. DPH (kcal/mol)	Average ∆G_4_ w.r.t. DPH (kcal/mol)	∆∆G_4-1_ w.r.t. DPH (kcal/mol)	IC_50_ (µM)*
DPH	0	0	0	1.99
3-MXP	1.90 ± 0.2	1.25 ± 0.5	−0.65 ± 0.3	0.587
4-MXP	2.64 ± 0.1	1.89 ± 0.5	−0.75 ± 0.4	2.23
2-Cl-DPH	−7.19 ± 0.2	−6.84 ± 0.7	0.35 ± 0.5	10.5
2-MXP	11.96 ± 0.2	15.59 ± 0.8	3.63 ± 0.6	30

## Supplementary Materials

The following are available online at http://www.mdpi.com/2076-3425/8/4/63/s1, Table S1: The convergence of the calculated free energy change (kcal/mol) in each perturbation for the five compounds in the bound (protein-ligand complex in water) state; Figure S1: Alignment of human (hDAT), rat (rDAT), mouse (mDAT) and fruit-fly (dDAT) amino acid sequences; Figure S2: (A) RMSD graph and superimposition of compounds at the binding site after docking (cyan) and after 100ns (orange) of unbiased MD for (B) DPH and (C) 2-MXP; Figure S3: Time evolution in the simulations for rDAT when bound to 2-Cl-DPH, Figure S4: Time evolution in the simulations for rDAT when bound to 3-MXP, Figure S5: Time evolution in the simulations for rDAT when bound to 4-MXP.
