# Supplementary material for: Mechanistic Insights into the Stimulant Properties of Novel Psychoactive Substances (NPS) and Their Discrimination by the Dopamine Transporter—In Silico and In Vitro Exploration of Dissociative Diarylethylamines
Source: Brain Sci. 2018 Apr 7;8(4):63. doi: 10.3390/brainsci8040063 (PMC5924399; doi:10.3390/brainsci8040063)
Supplement: Supplementary file 1 [file brainsci-08-00063-s001.pdf]

SUPPLEMENTAL INFORMATION

Mechanistic insights into the stimulant properties of novel psychoactive substances (NPS) and their discrimination by the dopamine transporter – *in silico* and *in vitro* exploration of dissociative diarylethylamines

Michelle A. Sahai[1], Colin Davidson[2,3], Neelakshi Dutta[2], and Jolanta Opacka-Juffry[1*]

[1]Department of Life Sciences, University of Roehampton, London SW15 4JD

[2]St George's University of London, SW17 0RE

[3]Pharmacy & Biomedical Sciences, University of Central Lancashire, Preston PR1 2HE, UK

*Correspondence:

Professor J. Opacka-Juffry

Department of Life Sciences

University of Roehampton, London SW15 4JD, UK

Tel +44 (0)20 8392 3563

Email: j.opacka-juffry@roehampton.ac.uk

Table S1. The convergence of the calculated free energy change (kcal/mol) in each perturbation for the five compounds in the bound (protein-ligand complex in water) state. (N.B. This is not the change of the binding free energy). The error was estimated based on the standard deviation of all the values used in the estimation of the final value.

Compound	ΔG_4 w.r.t. DPH (kcal/mol) 100ns	ΔG_4 w.r.t. DPH (kcal/mol) 150ns	ΔG_4 w.r.t. DPH (kcal/mol) 175ns	Average ΔG_4 w.r.t. DPH (kcal/mol)
DPH	0	0	0	0
3-MXP	1.19	1.25	1.28	1.25 ± 0.5
4-MXP	1.84	1.89	1.92	1.89 ± 0.5
2-Cl-DPH	-6.77	-6.80	-6.84	-6.84 ± 0.7
2-MXP	15.51	15.59	16.02	15.59 ± 0.8

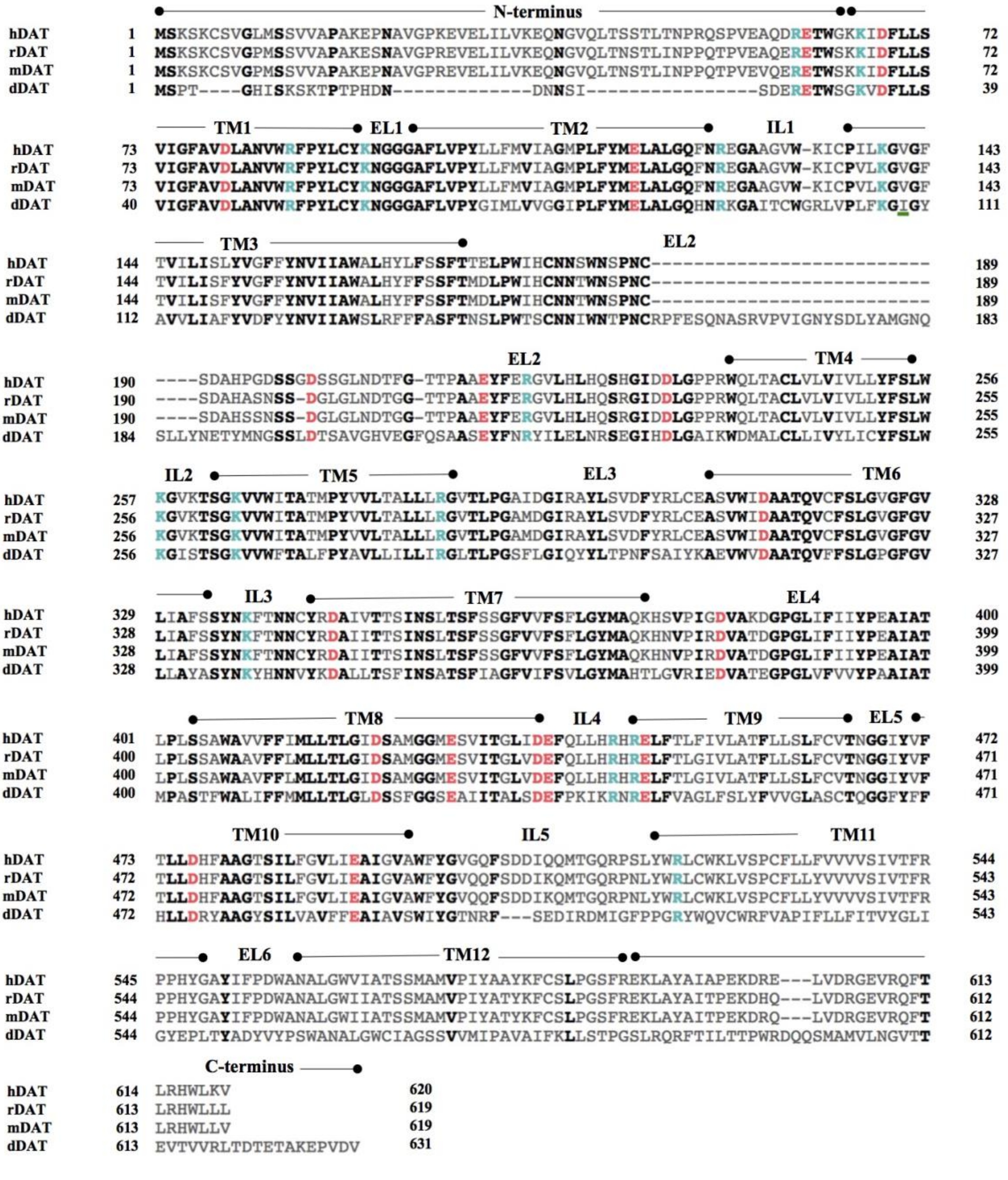

Figure S1: Alignment of human (hDAT), rat (rDAT), mouse (mDAT) and fruit-fly (dDAT) amino acid sequences. Conservation > 99% is indicated in black and highlights the high conservation at the transmembrane (TM) regions, especially TM1-10. Positively charged residues are coloured in red and negatively charged residues in cyan. The figure was prepared using Alignment-Annotator web server [42].

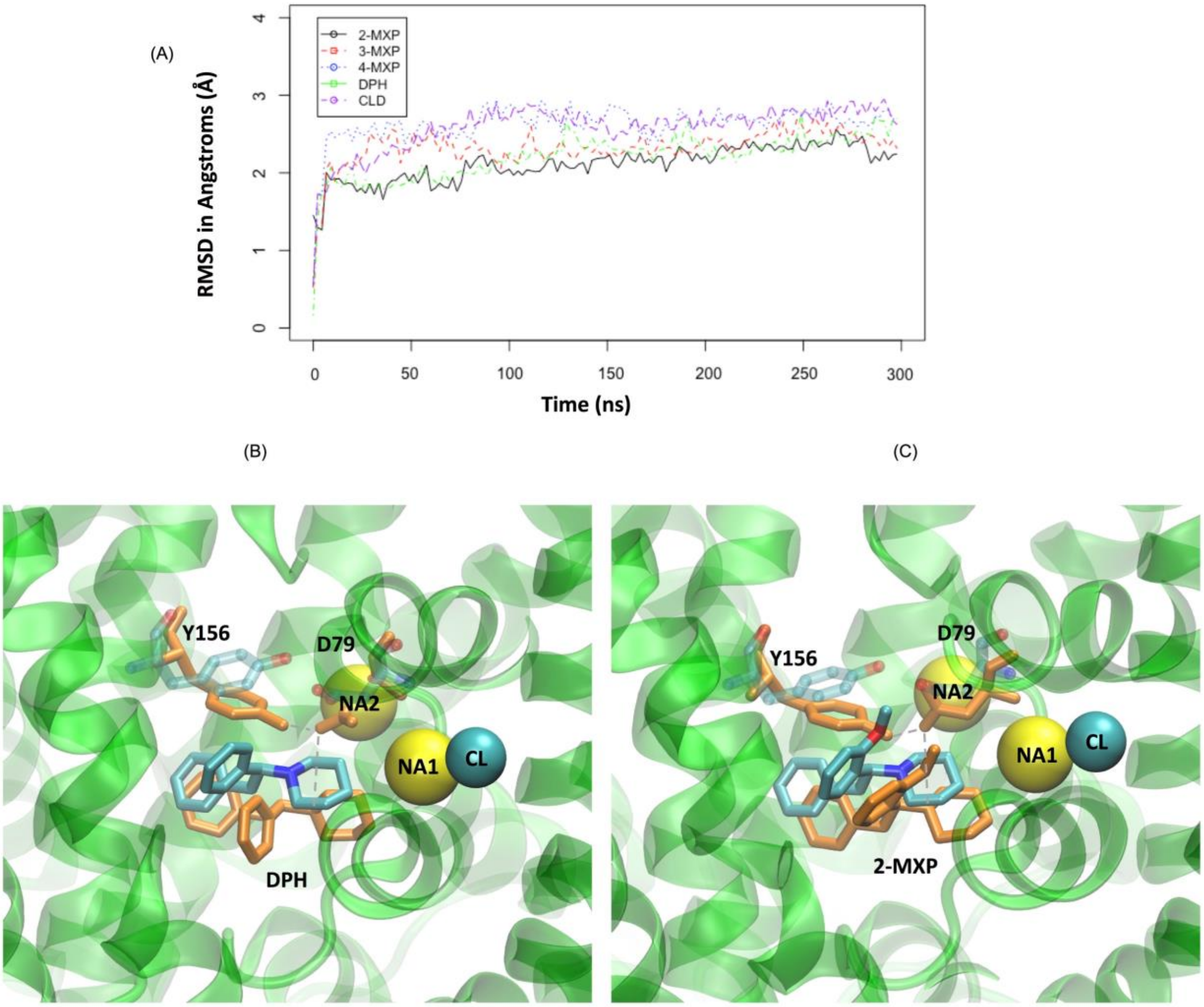

Figure S2: (A) RMSD graph and superimposition of compounds at the binding site after docking (cyan) and after 100ns (orange) of unbiased MD for (B) DPH and (C) 2-MXP. Internal sodium and chloride ions are depicted as yellow and cyan coloured spheres. Dashed lines refer to the salt bridge interaction between Asp79 and the protonated amine of the respective compound as well as the hydrogen bond between the OH group of Tyr156 and Asp79.

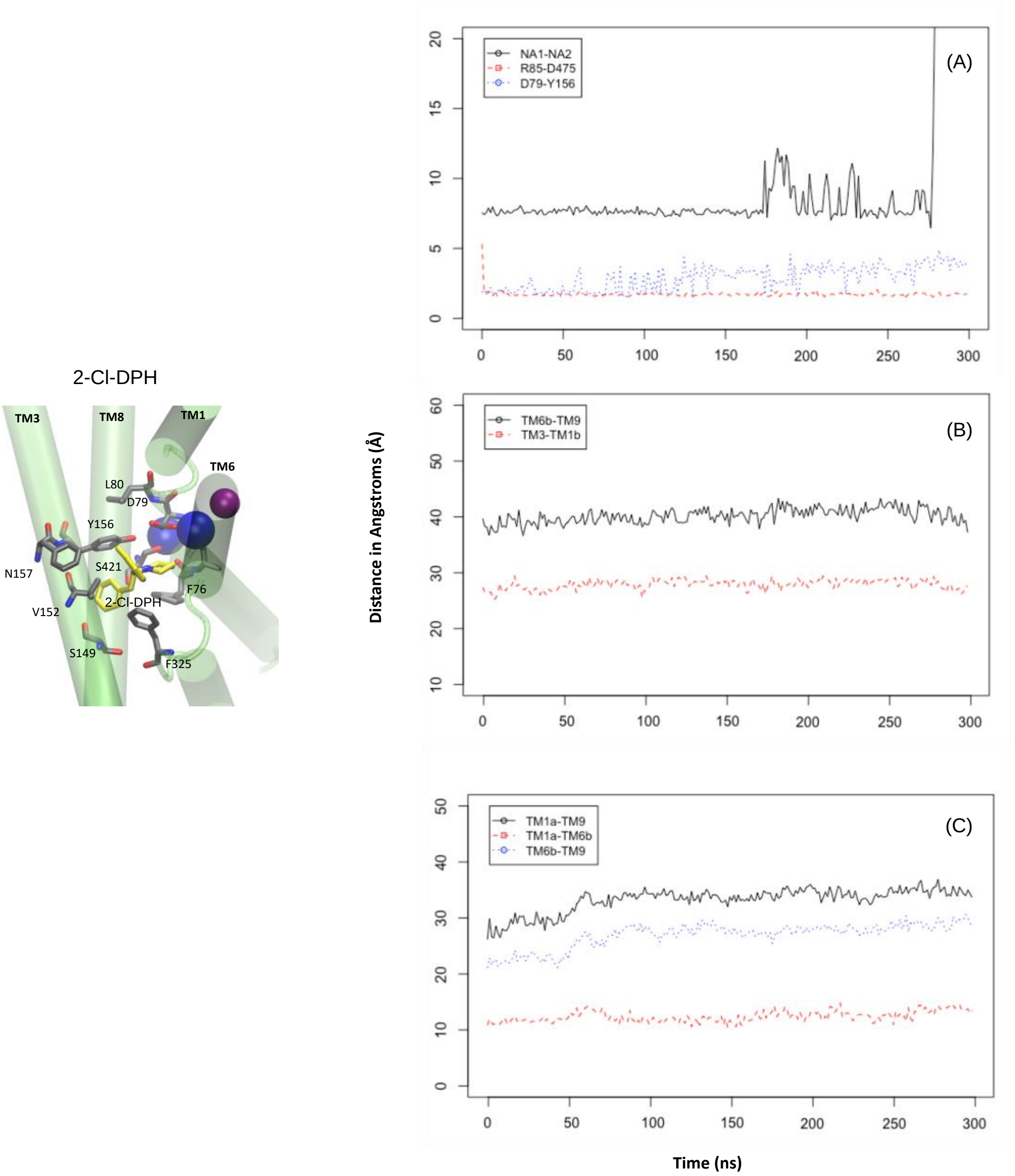

Figure S3: Time evolution in the simulations for rDAT when bound to 2-Cl-DPH. (A) depicts the distances between D79 and Y156, Na1 and Na2 and R85 and D475. (B) and (C) depict the Cβ-Cβ distances between residues in various extracellular and intracellular transmembrane (TM) segments respectively for I67 (in TM1a) and L446 (in TM9), I67 (in TM1a) and S332 (in TM6b) and S332 (in TM6b) and L446 (in TM9); E306 (in TM6a) and F171 (in TM3); and F171 (in TM3) and K92 (in TM1b).

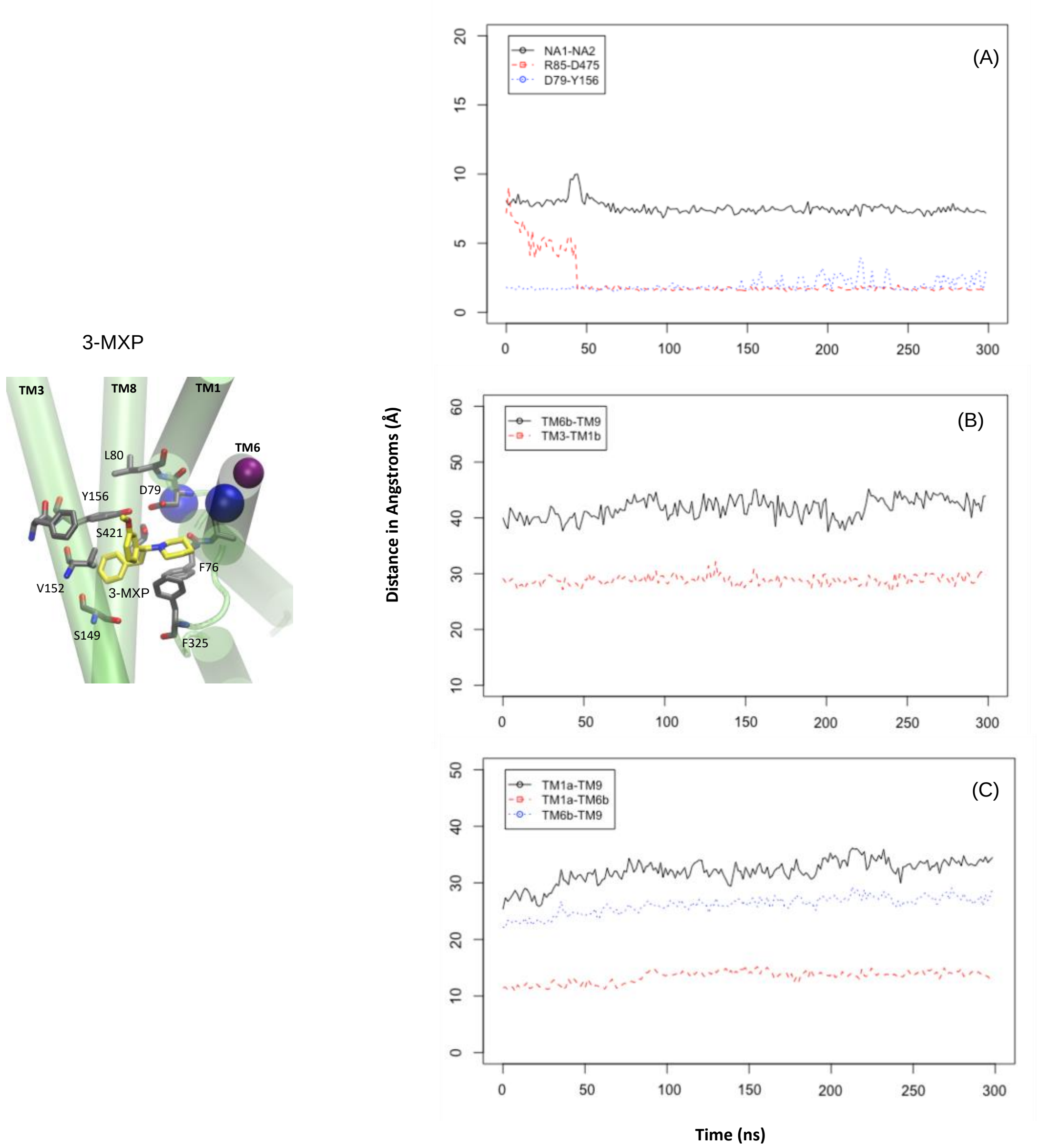

Figure S4: Time evolution in the simulations for rDAT when bound to 3-MXP. (A) depicts the distances between D79 and Y156, Na1 and Na2 and R85 and D475. (B) and (C) depict the Cβ-Cβ distances between residues in various extracellular and intracellular transmembrane (TM) segments respectively for I67 (in TM1a) and L446 (in TM9), I67 (in TM1a) and S332 (in TM6b) and S332 (in TM6b) and L446 (in TM9); E306 (in TM6a) and F171 (in TM3); and F171 (in TM3) and K92 (in TM1b).

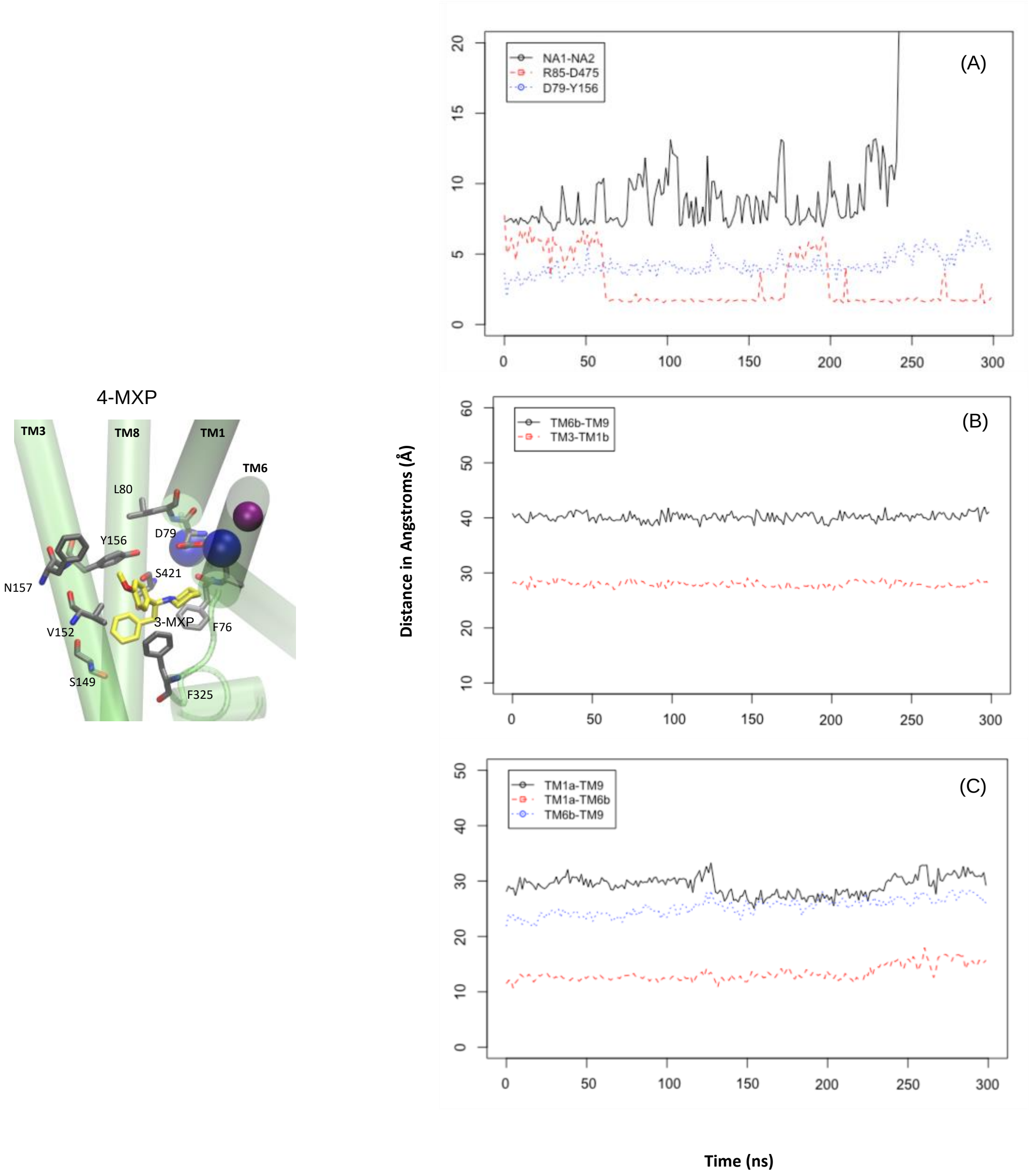

Figure S5: Time evolution in the simulations for rDAT when bound to 4-MXP. (A) depicts the distances between D79 and Y156, Na1 and Na2 and R85 and D475. (B) and (C) depict the Cβ-Cβ distances between residues in various extracellular and intracellular transmembrane (TM) segments respectively for I67 (in TM1a) and L446 (in TM9), I67 (in TM1a) and S332 (in TM6b) and S332 (in TM6b) and L446 (in TM9); E306 (in TM6a) and F171 (in TM3); and F171 (in TM3) and K92 (in TM1b).
